# Miniaturized Bidirectional Thermal Stimulation System Integrated With an Electrode Array for Recording Neural Activities

**DOI:** 10.1002/advs.202522077

**Published:** 2026-05-20

**Authors:** Zoia Naumkina, Kanghwan Kim, Wesley Charles Smith, Keuntae Kim, Shinwoo Lee, Jiwan Woo, Il‐Joo Cho

**Affiliations:** ^1^ Department of Biomedical Sciences College of Medicine Korea University Seoul Republic of Korea; ^2^ Brain Science Institute Korea Institute of Science and Technology (KIST) Seoul Republic of Korea; ^3^ Department of Convergence Medicine College of Medicine Korea University Seoul Republic of Korea; ^4^ Research Animal Resource Center Korea Institute of Science and Technology (KIST) Seoul Republic of Korea

**Keywords:** bidirectional stimulation, electrophysiological activity, microelectrode array, thermal neural modulation

## Abstract

Investigating the neural mechanisms underlying brain function is essential for advancing both our understanding of neurological processes and the development of effective treatments. However, traditional neuromodulation techniques typically provide only unidirectional control—either excitation or inhibition–limiting their capacity to investigate dynamic brain circuits. Here, we present a miniaturized, bidirectional thermal stimulation system integrated with a multichannel electrode array, enabling real‐time, bidirectional modulation through localized heating and cooling. This innovative system allows precise and reversible control of brain temperature, facilitating both excitation and inhibition within a single, compact platform. We demonstrate its capability by targeting the locus coeruleus (LC) in head‐fixed mice, inducing robust, directionally dependent changes in neural activity and accompanying behavioral responses, such as pupil dilation and constriction. In contrast to conventional approaches, our system achieves high temporal resolution and seamless integration of stimulation and recording. The proposed system provides a powerful and versatile tool for dissecting the dynamic regulation of neural circuits and opens new avenue for developing interventions for neurological and neurodegenerative disorders.

## Graphical abstract

This study presents a miniaturized bidirectional thermal stimulation system integrated with an electrode array enabling real‐time, bidirectional modulation and simultaneous recording of neural activity through localized heating and cooling. By monolithic integration of the Peltier element and with a silicon neural probe, the innovative system allows precise and reversible control of brain temperature, facilitating both neural excitation and inhibition within a single, compact platform.

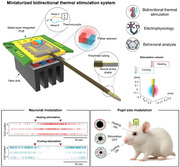

## 1 Introduction

Significant efforts to understand the functional neural circuits and develop treatments for their disorders through neuromodulation have driven the development of neural implants. Each new generation of neural probe technology has advanced toward achieving higher spatial and temporal resolution, integrating larger electrode arrays, and ensuring reliable long‐term recordings [1, 2, 3, 4, 5, 6].

However, for the study of brain functions and treatment of brain diseases, it is critical to go beyond simple neural recordings by integrating stimulation capabilities. Until now, electrical stimulation has been the widely used method of neuromodulation, especially in clinical purposes in the past two decades [7]. This type of modulation can effectively activate neural circuits but is limited in inhibiting neuronal activity due to its unidirectional nature [8, 9]. Optogenetic stimulation, on the other hand, utilizes genetically encoded opsins to precisely target specific neurons and offers both activation and inhibition capabilities. However, co‐expression of multiple opsins to achieve bimodal modulation may be technically challenging [10]. To overcome the need for genetic modification, several non‐genetic optical neuromodulation techniques have been proposed [11, 12]. Although these systems enable bidirectionality, they are typically implemented as planar thin‐film interfaces and are therefore mainly limited to surface or shallow neural stimulation.

As an alternative approach, we can modulate neural activities by injection of neurotransmitters or neuromodulators; however, precise control over chemical agent delivery is constrained by diffusion and spatial limitations, resulting in poor spatial and temporal resolution [13]. Although these methods offer therapeutic promise, the demand for innovative strategies that facilitate precise and localized neural modulation in deep brain regions continues to grow.

In response to this need, recent studies on thermal stimuli offer a novel avenue for neural stimulation, expanding the toolbox for brain research and therapy. This technique involves applying controlled temperature changes to specific brain regions to regulate neural activity and examine their functional consequences. Several groups have conducted studies demonstrating that temperature shifts can successfully modulate neural activity [14, 15, 16]. Liquid circulators were typically used to regulate surface brain temperature by circulating saline or antifreeze solutions within a cranial chamber [17]. Other studies have introduced a head‐mounted saline tank connected to the titanium cooling device to achieve more localized brain cooling [18]. Further achievements have included the use of artificial cerebrospinal fluid perfusion [19], integration of a U‐shaped duralumin plate [20], and the implantable microfluidic channels for peripheral nerve cooling‐based neuromodulation [21]. While these methods effectively inhibited neural activity, they are confined to the cortical surface and unsuitable for long‐term chronic use.

To achieve more precise stimulation with higher spatial resolution, Peltier elements have emerged as a promising tool, offering rapid temperature modulation through a simple thermoelectric principle. These elements create a temperature differential between two surfaces when an electric current is applied. Previous studies have introduced Peltier‐based thermal modulation system with applications ranging from direct contact with brain tissue to non‐invasive approaches through the scalp [22, 23, 24]. However, despite the Peltier system's ability to both cool and heat, the studies discussed above primarily focused on surface applications, limiting their ability to target deep brain regions.

Other groups have reported implantable systems for modulating temperature in localized deep brain regions. One system used a 330 µm gold wire encapsulated in silicone elastomer, connected to a Peltier element with a water‐cooled heat sink implanted in the songbird forebrain [25]. Another employed a silver wire sealed within a 1.1 mm polyimide tube, coupled with a Peltier element to directly stimulate neurons in the hippocampus [26, 27]. While these systems demonstrated the potential for localized deep brain stimulation, their bulky structure increased cross‐sectional areas and risked significant tissue damage, limiting their practicality for chronic implantation.

While the ability to stimulate is essential, recording neural activity is equally important for correlating modulation effects with neuronal responses. Notably, most temperature manipulation devices have been physically separated from the recording electrodes, posing challenges in placing the recording sites sufficiently close to the stimulation area. This separation not only reduces the precision of modulation‐recording correlation but also increases the extent of tissue damage due to the larger implant size. Therefore, it is important to integrate both thermal stimulation and neural recording in a compact, unified platform that enables bidirectional modulation while minimizing tissue disruption.

In this study, we report a miniaturized bidirectional thermal stimulation system integrated with an electrode array for simultaneous neural recording. We achieved monolithic integration of a thermoregulation modulator utilizing the Peltier effect with a silicon neural probe, chosen for its high thermal conductivity. To minimize heat dissipation, the probe was enclosed in a narrow polyimide tube, which acts as a thermal insulator by preserving an air gap. This design ensures a small footprint, efficient heat transfer, and precise thermal control in deep tissue. Additionally, polymer beads were used to center and stabilize the probe within the tube, maintaining equidistant spacing from the walls and optimizing thermal efficiency. This configuration was successfully validated using a thermochromic hydrogel to visualize temperature distribution.

By applying bidirectional current, our system enables both excitation and inhibition of neural circuits – a capability that distinguishes it from conventional unidirectional modulators. This was confirmed through simultaneous neural recording, offering a more comprehensive understanding of circuit dynamics. In particular, we demonstrated that bidirectional modulation of a locus coeruleus (LC) not only successfully modulated neural activity in a targeted region but also evoked measurable behavioral responses, such as pupil dilation and constriction, in lightly anesthetized, head‐fixed mice.

We believe that our miniaturized fully integrated thermal neuromodulation platform represents a groundbreaking advancement in neuromodulation technology. It offers precise, reversible stimulation and concurrent recording, with strong potential for both basic neuroscience research and therapeutic applications targeting neuropsychiatric and neurodegenerative disorders.

## 2 Results

### 2.1 Design, Fabrication, and Packaging of the Bidirectional Thermal Neural Probe

**FIGURE 1:**
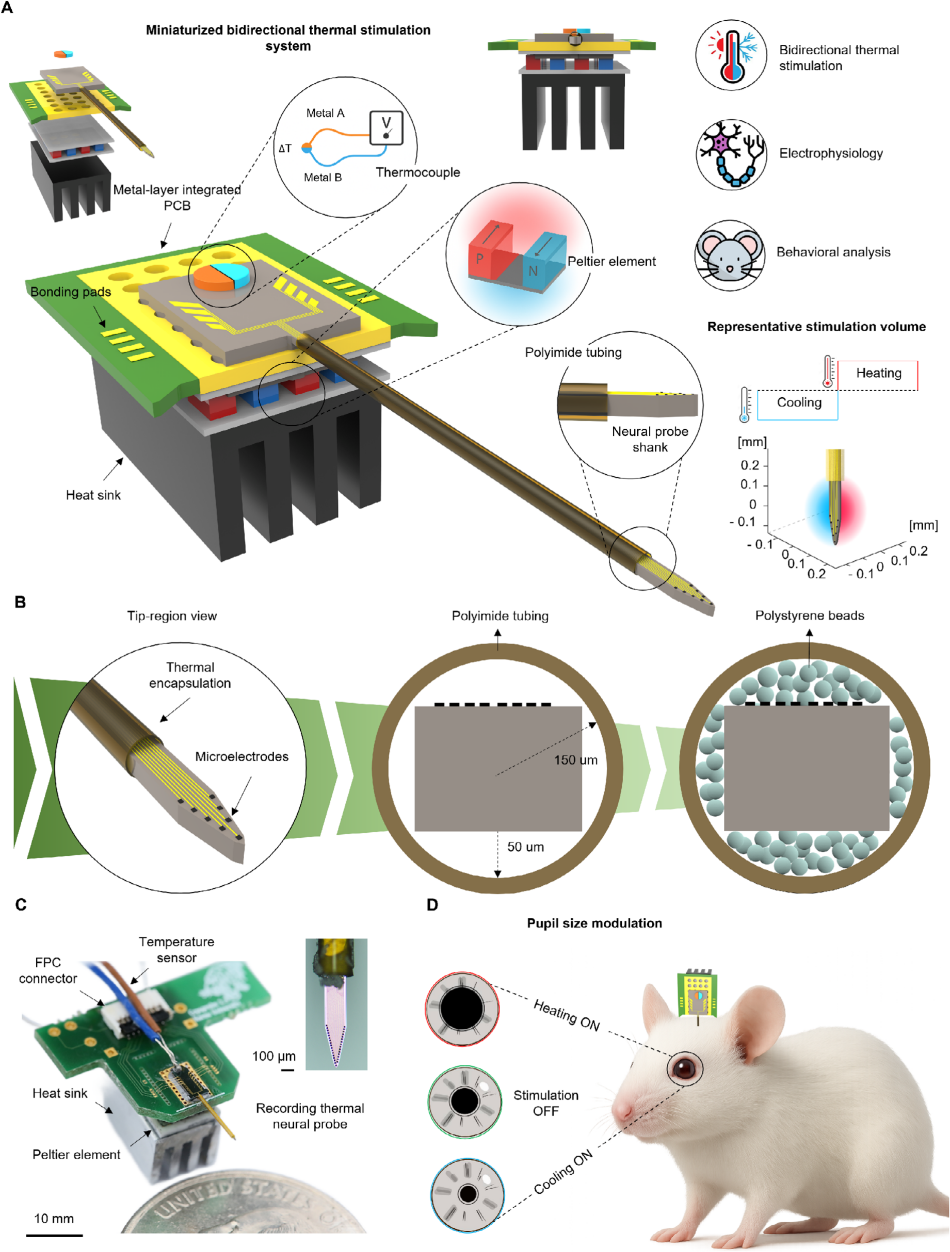
Design and operation principles of the bidirectional thermal stimulation system. (A) Schematic illustration of the miniaturized bidirectional thermal stimulation system characterized by representative stimulation area and its capabilities for bidirectional thermal stimulation and behavioral electrophysiology. (B) Tip‐region view of the neural probe shank encapsulated in polyimide tubing with integrated microelectrodes and polystyrene beads sealing. (C) Photograph of the overall configuration of the thermal stimulation system. (D) Schematic demonstration of in vivo functionality, showing bidirectional pupil modulation as a representative behavioral readout of thermal stimulation.

The miniaturized bidirectional thermal stimulation system was developed by integrating three key components into a single platform: (1) a bidirectional thermoelectric stimulator paired with a heat sink for controlled temperature generation; (2) an encapsulated probe shank, designed for targeted deep brain thermal modulation; and (3) a metal‐integrated printed circuit board (PCB) for electrical connection, ensuring efficient thermal transfer to the probe body (Figure 1). The probe array was fabricated based on a previously reported neural probe from our group [4, 28], with modifications to the shank's cross‐sectional area to improve axial thermal conduction (Figure S2).

The design of the proposed neural probe enables the probe structure to function as both temperature stimulation tool and recording element, with electrodes integrated onto its surface. To achieve accurate and efficient bidirectional temperature modulation, the system is designed to deliver thermal energy from the Peltier element which is mounted on the body of the neural probe to tip with minimal energy loss. When a current is applied, the Peltier device induces a temperature differential, producing simultaneous heating on one side and cooling on the other. To enhance thermal stability, a heat sink was mounted onto the Peltier element to dissipate excess heat into the environment, enabling stable and fast operation during heating and cooling cycles. During operation, thermal flux is transferred from the Peltier device directly to a metal‐layer‐embedded PCB of matching size. A copper layer, selected for its excellent thermal conductivity, ensures even distribution of temperature change across the PCB surface.

To ensure efficient thermal transfer, a layer of thermal paste was applied between the PCB matrix and the neural probe body. This interface improves thermal contact and promotes effective heat propagation from the metal vias to the silicon (Si) probe. The silicon substrate, with its high thermal conductivity, enables efficient axial transmission of temperature along the probe shank and establishes a stable gradient toward the tip. To minimize heat loss and localize the area of stimulation, the probe was enclosed in a hollow polyimide tube with, only the probe tip exposed for direct interaction with brain tissue during thermal modulation and neural signal acquisition.

The neural probe was fabricated with a single‐shank configuration (6.0 mm in length, 190.0 µm in thickness), allowing access to the full depth of an adult mouse brain. The specific thickness allows for efficient axial heat conduction along the shank, ensuring that temperature changes at the base propagate effectively to the probe tip. The device includes an array of 32 microelectrodes (14 × 14 µm each) for recording neural activity surrounding the probe during stimulation cycles (Figure 1A). To enhance recording performance, the electrodes were electroplated with platinum black (Figures S3 and S4) [29]. The long‐term stability of the neural probe, including the platinum black‐electroplated microelectrodes, has been previously validated by our group [30]. In this configuration, the silicon‐based neural probe serves as both a robust neural interface and a thermal conduit.

As previously described, to achieve precise thermal control in the deep brain regions, the shank was enclosed in a polyimide tube (5.5 mm length, 325.0 µm diameter). Polyimide was chosen for its superior insulation, biocompatibility, and low thermal conductivity (0.3 W/(m·K)) [31, 32]. To prevent direct contact between the probe and tube, polystyrene microbeads (thermal conductivity: 0.338 W/(m·K)) were packed into the gap, introducing air insulation and minimizing racial heat loss (Figure 1B). A thermal epoxy seal was applied to secure the microparticles in place, ensuring uniform spacing between the probe and tube wall, and maintaining consistent thermal insulation along the shank.

Thermal modulation was driven by a miniaturized thermoelectric Peltier module (6.2 × 6.2 × 2.68 mm) and passive heat sink (12 × 6.2 × 6.2 mm). The Peltier element comprises alternating *n*‐ and *p*‐type semiconductor materials forming thermoelectric junctions. Upon current application, the Peltier effect causes one side to absorb heat (cool side) and the other to releases it (hot side). By reversing current direction, the device can switch between heating and cooling modes within a single experimental session. To support thermal regulation, a heat sink made of lightweight aluminum (thermal conductivity ∼237.0 W/(m·K)) was attached to the back of the Peltier element, facilitating rapid dissipation of excess heat and maintaining system stability.

To monitor temperature in real time, a miniature thermocouple was integrated into the system. Based on the Seebeck effect, the thermocouple generates voltages proportional to the temperature difference between junctions, enabling continuous temperature tracking and closed‐loop thermal control throughout experiments.

The probe was attached to a custom‐designed printed circuit board (PCB) that accommodates both the electrical interface and thermal structure (Figure 1C). A metal matrix embedded within the PCB includes a copper base layer overlaid with gold, promoting efficient heat transfer (Figure S5). A via structure connects all layers vertically, facilitating uniform thermal conduction. The copper layer (thermal conductivity: ∼315.0 W/m·K)) transfers heat from the Peltier to the probe base, while the shank further conducts it to the tip (k = 125.0 W/m·K). This co‐integration of thermal stimulation and neural recording components results in a compact device (∼1 cm^3^ volume, 2.1 g mass). This integrated system is particularly suitable for targeting the locus coeruleus (LC) in the mouse brain, a region involved in regulating pupil dynamics (Figure 1D).

In summary, the proposed thermal neuromodulation system represents a substantial advancement in the precision neural interfacing. Its compact and lightweight design, efficient thermal architecture, and stimulation‐recording capability enable bidirectional thermal modulation of deep brain circuits with high spatiotemporal control, offering a powerful tool for dissecting neural activity dynamics with minimal behavioral disruption.

### 2.2 Characterizations of the Bidirectional Thermal Neural Probe

**FIGURE 2:**
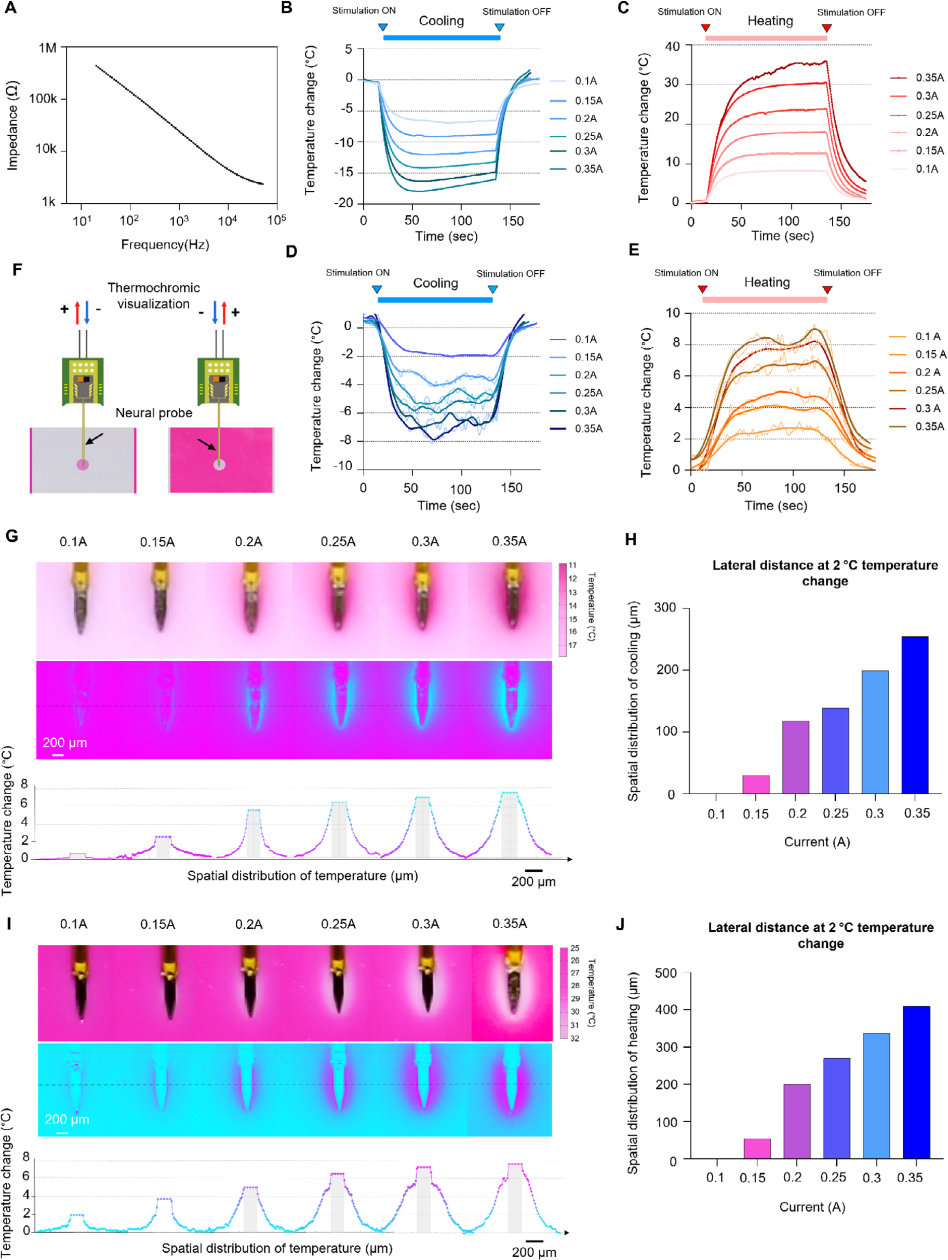
Characterization of the bidirectional thermal neural probe. (A) Impedance plot of the platinum black (Pt Black) microelectrodes. (B,C) Temperature change profiles measured from the body of the neural probe during cooling (B) and heating (C) cycles across various input currents (0.1–0.35 A). (D,E) Corresponding temperature change profiles measured at the tip of the neural probe during cooling (d) and heating (e). F, Schematic illustration of the thermochromic hydrogel visualization setup used to validate localized heating and cooling effects around the probe tip. (G) Sequential thermal imaging of the neural probe during the cooling process. Top panel: Optical images of the probe placed in thermochromic gel under varying currents. Middle panel: Colorimetric thermal maps derived from the gel, where blue indicates localized cooling and pink represents baseline or warmer regions. Bottom panel: Spatial temperature profiles extracted along the horizontal dashed line, quantifying lateral temperature gradients for each current amplitude. (H) Quantification of spatial cooling extent based on the lateral distance where temperature change reaches 2°C, demonstrating current‐dependent expansion of the affected area. (I) Heating characteristics of the neural probe visualized in thermochromic gel. Top panel: Optical images showing the probe surrounded by heat‐activated magenta regions under increasing current. Middle panel: Colorimetric maps where magenta indicates heating and blue represents baseline temperature. Bottom panel: Spatial temperature profiles extracted along the dashed line, showing both peak temperature rise and lateral expansion of heat with stronger stimulation. (J) Quantification of spatial heating extent measured at the 2°C threshold, revealing a proportional increase in temperature spread with higher stimulation currents.

Prior to in vivo experiments, we evaluated the performance of the proposed bidirectional thermal neural probe by characterizing three key aspects: (1) the electrical impedance of the electrodes, (2) temporal control of temperature modulation at the probe body and tip, and (3) spatial distribution and localization of thermal stimulation. First, we measured the electrical impedance of the 32 microelectrodes both before and after Pt black electroplating. The average impedance measured at 1 kHz was approximately 25.0 kΩ, a sufficiently value to ensure reliable neural signal recording (Figure 2A).

Second, we examined the spatiotemporal temperature dynamics at the probe body at various current levels using the integrated thermocouple (Figure S6). The thermal modulation curves during cooling and heating cycles (Figures 2B‐C) demonstrated that our system could steadily and precisely reach target temperatures. Cooling to a 20.0°C was achieved within ∼20 s at 0.35 A (1.4 V), while heating to 30.0°C was reached at 0.30 A, confirming fast response and strong thermal controllability.

To quantify the actual thermal change at the exposed tip, we repeated the same current protocols and measured the temperature using a miniaturized thermocouple placed at the tip (Figure 2D,E; Figure S6). The temperature profiles closely mirrored those at the probe body but exhibited slightly attenuated amplitudes, with a cooling of ∼8.0°C and heating of ∼8.0°C at ±0.35/0.3 A, respectively. This difference likely results from intrinsic thermal resistance along the probe and passive dissipation into surrounding media, even with the polyimide tube minimizing lateral heat loss.

Neural tissue is known to tolerate transient temperature increases up to 45°C and decreases down to ∼30°C without incurring damage within a 10 min exposure window [33, 34]. All operating ranges of our system remain within these safety margins, supporting the feasibility of sustained, adjustable thermal neuromodulation without thermal injury.

Localized control of temperature at the probe tip is a critical feature of our system. To evaluate this capability in a physiologically relevant context, we employed thermochromic hydrogel (Insilico Co. Ltd., South Korea) that change color at specific temperatures to visually map the spatial extent of thermal modulation.

Using thermochromic slurries tuned to color‐shift at 18.0°C and 25.0°C, we implemented agarose‐based hydrogel blocks for visualizing the cooling and heating zones, respectively (Figure 2f). The neural probe was carefully embedded into the hydrogel cube, and thermal spread was monitored in real‐time. This setup enabled clear visualization of the bidirectional thermal influence centered around the expose tip, confirming strong spatial confinement of the stimulation volume (detailed thermal mapping procedure is provided in the Methods section, Movies S1 and S2 and Figures S7–S11).

To evaluate the current‐dependent spatial spread of thermal modulation, we conducted a comprehensive analysis of temperature distributions during both cooling and heating cycles under various current amplitudes.

For cooling, a top‐down view of the neural probe embedded in thermochromic hydrogel is shown, accompanied by corresponding colorimetric maps where blue represents cooled regions and pink indicates baseline or warmer temperatures (Figure 2G, top and middle). Temperature values were extracted along the horizontal dashed line to generate spatial temperature profiles (Figure 2G, bottom). At the lowest current of 0.1 A, no noticeable color change was observed in the gel, indicating minimal or no effective cooling at this level. These results revealed that the most intense cooling (∼7.0°C–8.0°C) occurred immediately adjacent to the probe and gradually diminished with distance. As the applied current increased beyond 0.15 A, both the peak temperature drops and the lateral spread of the thermal effect became more prominent, indicating tunable and localized modulation capabilities.

To quantify this relationship, we measured the lateral distance at which the local temperature change reached 2.0°C (Figure 2H) and calculated the temperature drop at 200 µm from the probe center for each current level (Figure S12). These results confirmed a proportional increase in effective stimulation area with rising current amplitude.

For heating, we applied currents ranging from 0.1 to 0.35 A. The top‐down thermal images and associated colormaps show magenta‐shaded heated regions and blue baseline zones (Figure 2I, top and middle). The spatial temperature profiles (Figure 2I, bottom) revealed a peak temperature increase from ∼2.0°C at 0.1 A to ∼7°C–8°C at 0.3 A, with a ∼4.0°C reduction measured at 200 µm. The spatial spread of heating also expanded consistently with current amplitude (Figure 2J), confirming the symmetric and scalable nature of the thermal effect.

To consolidate the dynamic thermal behavior of the probe, we summarized temporal measurements from both the probe body and tip in unified plots (originally shown in Figure 2B,C). During cooling at 0.35 A, the tip temperature dropped by ∼8.0°C and returned to baseline within ∼150 s, while heating at 0.2 A led to a ∼4.0°C increase followed by a similar recovery period.

To further evaluate the thermal distribution generated by the thermoelectric probe within brain tissue, we performed finite‐element analysis (FEA) using COMSOL Multiphysics under operating conditions matching those to be used in the subsequent in vivo experiments (Figure S13). Under the cooling condition (0.35 A), the model predicted a tip temperature of approximately 29.0°C, corresponding to a temperature decrease of ∼9.0°C relative to physiological brain temperature. The simulation reproduced the spatial decay of cooling observed in the hydrogel measurements, with a temperature decrease of 5.8°C at a lateral distance of 200 µm from the probe center.

Under the heating condition (0.2 A), the simulation predicted a local temperature of ∼39.7°C near the probe tip, corresponding to an increase of 3.0°C above physiological brain temperature. The simulated temperature profiles showed rapid attenuation with distance from the probe, consistent with the spatial patterns observed in the thermochromic hydrogel experiments.

We also investigated the effect of implantation depth on thermal distribution using COMSOL simulations (Figures S14 and S15). Temperature profiles extracted as a function of distance from the probe tip enabled comparison of thermal behavior across different depths. Both cooling and heating effects remained spatially localized near the probe tip but became progressively attenuated with increasing insertion depth.

A depth‐dependent trend was observed at the probe tip when referenced to physiological brain temperature (∼37.5°C). For cooling, the tip temperature increased from ∼21.0°C at 0.7 mm to ∼27.0°C at 3.5 mm, corresponding to a reduction in cooling capacity of approximately 36.0%. In contrast, for heating, the tip temperature decreased from ∼40.5°C to ∼39.4°C over the same depth range, corresponding to a reduction in heating capacity of approximately 37.0%. Although the simulations were limited to 3.5 mm due to the physical length of the current probe, these results indicate that thermal attenuation increases with insertion depth, suggesting that further depth extension would likely lead to additional reduction in temperature modulation due to increased heat dissipation along the probe shaft and into the surrounding tissue.

To verify thermal modulation under physiological conditions, we performed in situ temperature measurements by co‐implanting an 80 µm‐diameter micro‐thermocouple with the stimulation probe in the brain tissue to validate thermal modulation under real physiological conditions (Figure S16). A 2 min stimulation protocol was applied for both heating and cooling conditions to allow the temperature to stabilize (Figure S17).

During heating at 0.2 A (1.4 V), the local brain temperature increased by approximately 3.7°C, in close agreement with both hydrogel measurements and finite‐element simulations, corresponding to an absolute temperature of ∼41°C. Increasing the current to 0.35 resulted in a temperature rise up to ∼44.5°C. During cooling at 0.35, the measured temperature decrease was approximately 3.9°C (corresponding to ∼33°C), representing a ∼35.0%–45.0% reduction compared to hydrogel measurements. This difference is attributed to continuous blood perfusion in the brain, which acts as a heat sink and partially compensates for the cooling effect.

The asymmetry between cooling and heating responses is consistent with perfusion‐mediated heat transfer, where warm blood more effectively offsets cooling than it suppresses localized heating. Minor variations may also arise from the spatial positioning of the thermocouple relative to the probe tip (∼20 µm separation).

Together, these findings validate the probe's ability to achieve steady, well‐controlled, and symmetric bidirectional thermal neuromodulation. The localized effects span approximately 200 µm laterally from the probe tip, enabling targeted neural modulation with minimal thermal spread beyond the intended area.

### 2.3 Bimodal Thermal Modulation of Neural Activities in Localized Brain Region In Vivo

**FIGURE 3:**
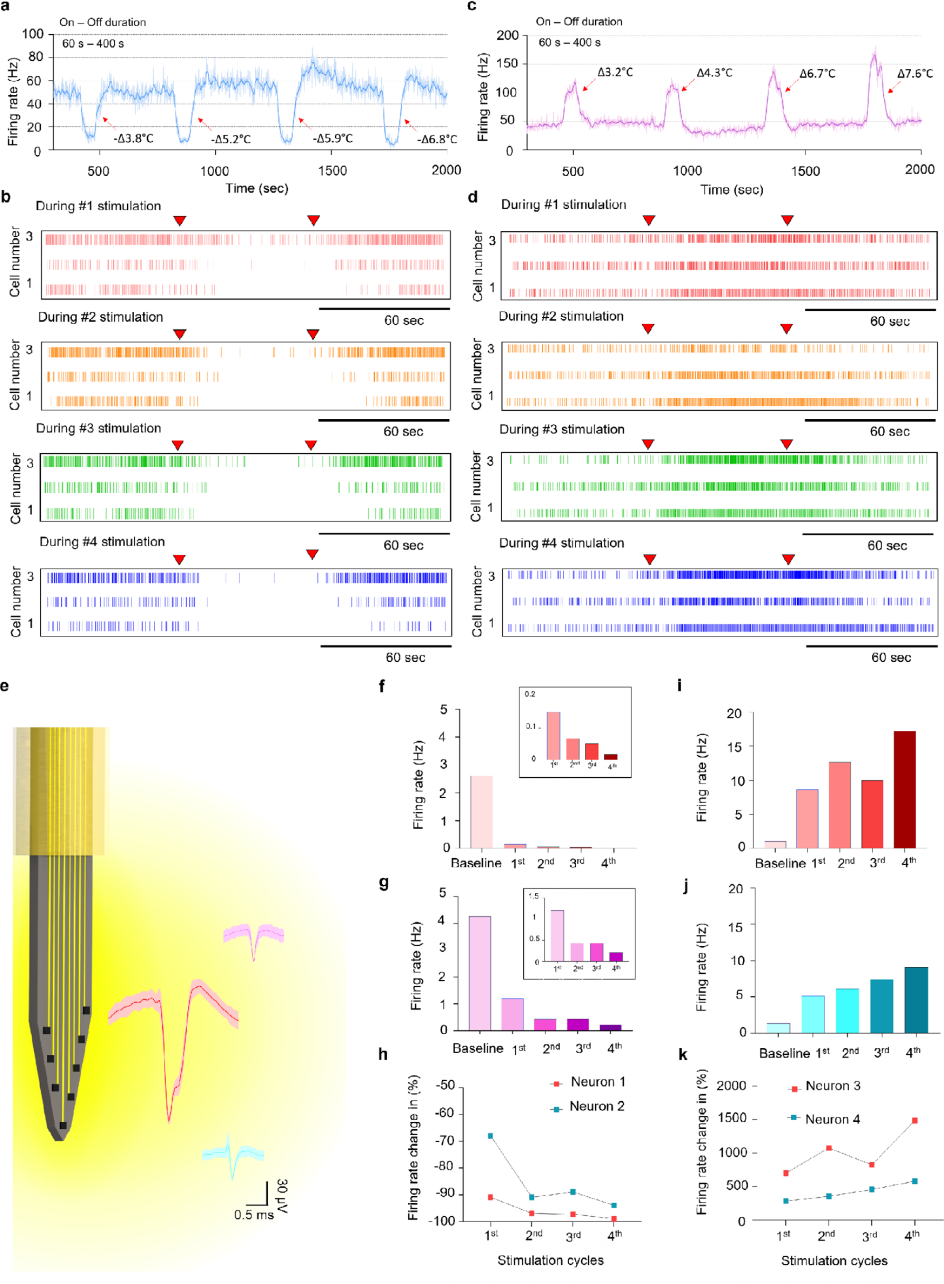
Temperature‐dependent modulation of the neural activity. (A) Series of firing rate traces showing the suppression of neuronal activity during sequential cooling cycles (60 s ON, 400 s OFF), with corresponding temperature changes (ΔT) values indicated. (B) Neural firing patterns of cerebellum neurons during four distinct cooling stimulations, each with increasing current amplitude and corresponding temperature decrease. (C) Heating‐induced increase in firing rates over sequential stimulation cycles with increasing ΔT across stimulation epochs. (D) Neural spike raster plot demonstrating the dose‐dependent excitation during the heating cycles with increasing current amplitudes and ΔT. (E) Schematic illustration of the neural probe system and representative extracellular spike waveforms recorded from nearby neurons. (F) Firing rate of the neuron closest to the probe during repeated cooling stimulations. Inset: magnified low‐rate responses. (G) Cooling‐induced firing rate of the neuron farthest from the probe. (H) Percent changes in firing rates across neurons during cooling stimulation cycles. (I) Firing rate of the neuron closest to the probe during heating stimulation. (J) Heating‐induced firing rate change of the neuron farthest from the neural probe. (K) Percent change in firing rate of two representative neurons across heating cycles. Cooling and heating stimulations were performed in 2 animals each (*n* = 2, where *n* is the number of animals).

Next, to validate the bimodal modulation of neural activities by thermal modulation, we implanted the neural probe into the cerebellum and recorded neural signals under both cooling and heating conditions. Controlled thermal cycles were applied to induce graded temperature changes at the target site (schematic illustration of the operation system is provided in the Figure S18). Following a 20 min stabilization period, stimulation protocols were initiated, consisting of alternating cooling and heating cycles. Each cycle included a 60 s stimulation phase followed by a 4 min rest period (Figure 3A,C).

The recorded neural signals revealed distinct temperature‐dependent modulation of firing rates in responses to both cooling and heating. During the cooling cycles (Figure 3A; Figure S19), multi‐unit firing rates progressively decreased as the degree of temperature reduction increased. Specifically, cooling by 3.8°C and 6.8°C resulted in 22.7% and 8.0% of the baseline firing rate, respectively (Figure S20). These effects were clearly reflected in the raster plots, which demonstrated stronger inhibition during deeper cooling cycles, as indicated by red arrows. This highlights the robust suppressive influence of low temperatures on neuronal excitability.

Conversely, during heating cycles (Figure 3C), neuronal activity increased substantially in a temperature‐dependent manner. Raising the probe temperature by 3.2°C to 7.6°C resulted in firing rate elevations to approximately 199.0% and 322.0% of baseline, respectively. The excitatory effect peaked several seconds after the onset of heating and gradually returned to baseline during the following rest period, illustrating the transient and reversible nature of thermal excitation. Overall, heating cycles resulted in a pronounced increase in firing rates, showing dose‐dependent excitation in response to elevated temperatures (Figure 3D). The cumulative results across multiple cycles are summarized in Figure 3D, showing a consistent trend of dose‐dependent excitation with increasing temperature.

The cooling periods demonstrated a clear inhibitory effect on neuronal firing rates, with the degree of suppression varying depending on the presumed distance of each neuron from the cooling site. Neurons inferred to be close proximity to the probe – based on their larger spike amplitude (e.g., 180 µV) – exhibited a rapid and substantial decline in the firing rate, even with modest cooling (Figure 3F). This immediate suppression of the firing rate in these neurons suggests a high sensitivity to cooling, as further temperature decreases resulted in only slight additional reduction in neural activity. For instance, a 3.8°C temperature drop reduced the firing rate to 7.0% of the baseline, and a 5.3°C drop further reduced it to approximately 1.0%. Notably, this rapid suppression plateaued despite further cooling, indicating high sensitivity and saturation of the inhibitory effect in nearby neurons.

By contrast, neurons presumed to be farther from the cooling site – indicated by smaller spike amplitude (e.g., 60 µV) – exhibited a more gradual response (Figure 3G,H). In these neurons, firing rates remained relatively stable during the initial cooling cycle but declined progressively with increasing thermal intensity. A 3.8°C decrease reduced the firing rate to 28.0% of baseline, while 5.3°C decrease further reduced it to 4.7%. This represents a ∼18.0% additional reduction compared to 5.0% further reduction observed in nearby neurons under the same cooling condition. This pattern likely reflects the spatial dynamics of thermal diffusion: neurons farther from the probe lie in regions where the cooling effect is weaker, requiring greater thermal change to induce significant inhibition. These results suggests that cooling generates a spatially confined “volume of inhibition” that expands with increasing temperature drop, selectively suppressing neurons based on their distance from the cooling source (Figure S21).

In contrast, during the heating periods, firing rates increased in both proximal and distal neurons (Figure 3I–K). However, continuously increased with temperature rise. Notably, the neuron presumed to be closer to the probe (larger amplitude, 180 µV) showed a larger firing rate increase – up to 700.0% of baseline after the first stimulation cycle (Figure 3I). The more distant neuron (smaller amplitude, 60 µV) also showed a substantial increase of ∼300.0%, though the magnitude of modulation was smaller (Figure 3J). Both neurons continued to show elevated firing rates with subsequent heating cycles (Figures S22 and S23).

Together, these results highlight the spatial selectivity of thermal neuromodulation: both cooling and heating create distance‐dependent changes in neural activity. This volume‐dependent modulation enables precise control over neuronal populations based on their relative location, offering a strategy for spatially targeted inhibition or excitation.

### 2.4 Bidirectional Modulation of Locus Coeruleus Activity and Pupil Dynamics Using a Thermal Neural Interface

This experimental design was intended to leverage the unique capabilities of our thermal neural interface – specifically, its ability to bidirectionally modulate neural activity while simultaneously recording electrophysiological signals and behavioral responses. This dual functionality allowed us to evaluate, within the same subject, how both inhibition and excitation of a single brain region influence downstream physiological outcomes. By applying thermal stimulation in both directions to the locus coeruleus (LC), a key neuromodulatory hub, we aimed to reveal the relationships between LC activity and autonomic behavioral readouts such as pupil dynamics. This within‐subject design not only minimized inter‐animal variability but also enables precise comparison of opposing neuromodulatory effects in a tightly controlled experimental framework.

**FIGURE 4:**
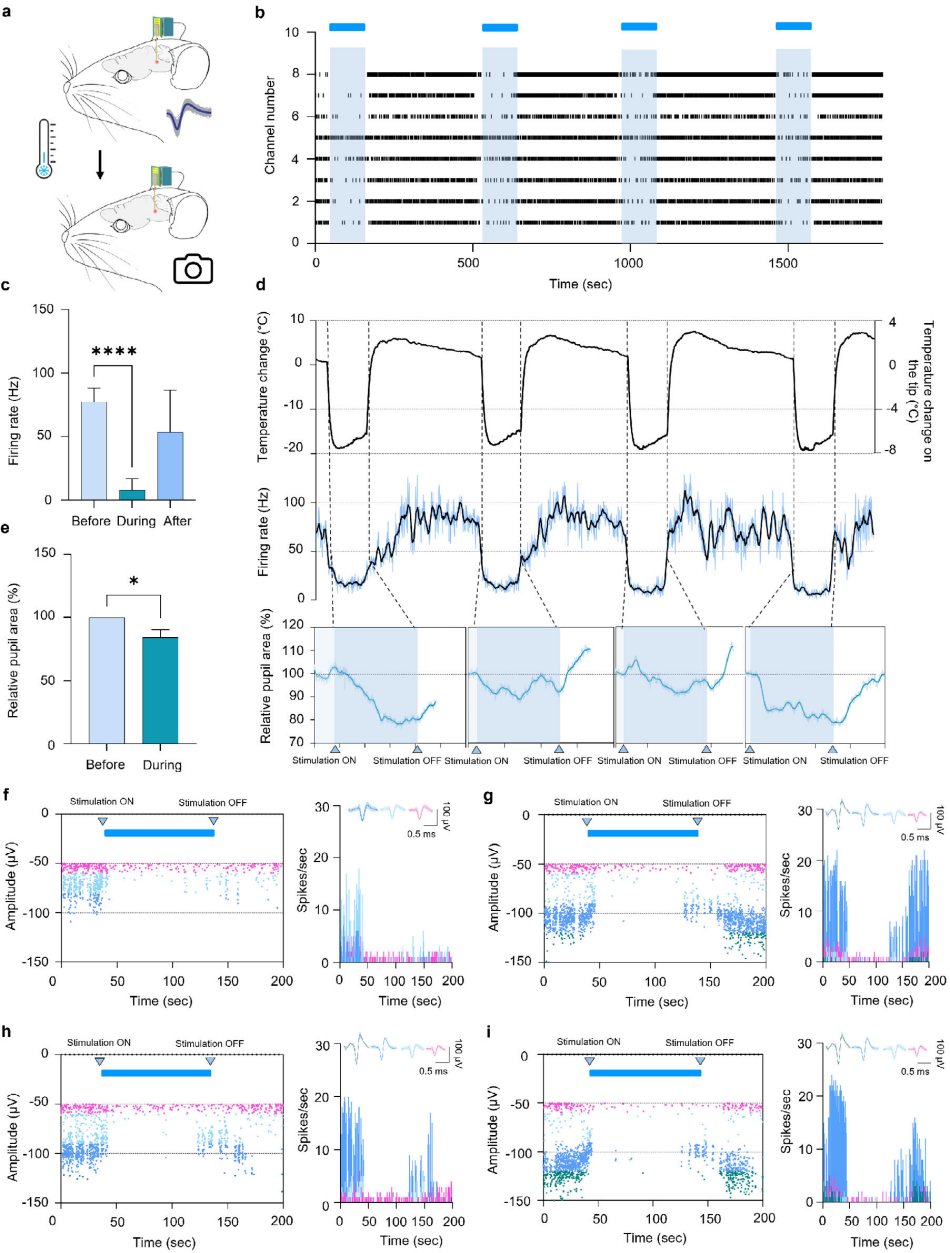
In vivo demonstration of cooling modulation capability of the miniaturized bidirectional thermal stimulation system. (A) Schematic of the experimental setup for in vivo cooling using the miniaturized bidirectional thermal stimulation system, with simultaneous pupil imaging and neural recording *(n* = 1, where n is the number of animals). (B) Raster plot of neuronal firing activity across multiple channels during cooling stimulation cycles (blue bars indicate stimulation periods). (C) Quantified average firing rates of LC neurons before, during, and after cooling stimulation (*p* = 0.0001; *n* = 4, where n is the number of stimulations, *t* = 24.36). (D) Simultaneous recordings of temperature change (top), LC firing rate (middle), and pupil area (bottom) over multiple stimulation cycles. (E) Pupil diameter changes showing pupil size before and constriction during cooling stimulation (*p* = 0.0137; *n* = 4, where n is the number of stimulations, *t* = 5.215. (F–I) Firing rates of representative LC neurons recorded during cooling stimulation cycles. Insets show representative spike waveforms. The blue line above the raster plot indicates the duration of cooling stimulation (3 cycles; 2 min ON and 4 min OFF per cycle). The inset shows representative neural signals. All statistical analyses were performed by the two‐tailed unpaired *t*‐test, and *p* < 0.05 was considered significant. ^*^ *p* < 0.05, ^**^ *p* < 0.01, ^***^ *p* < 0.001. ns: no statistical significance.

To achieve this, we utilized our bidirectional thermal modulation system, which supports simultaneous electrophysiological recording and behavioral monitoring. The neural probe was implanted into the LC of C57Bl6 wild‐type mice (Figure 4A) (detailed surgical procedure is provided in the Methods section).

The stimulation protocols consisted of alternating 2 min ON and 4 min OFF cycles. Spiking activity was recorded using custom MATLAB code (bandpass filter 0.3–6 kHz), while spontaneous was observed across all channels with a baseline noise levels of approximately ±15 µV. Following a 30 min stabilization period, we initiated localized LC inhibition via cooling conducted from the thermoelectric element on the probe body to the probe tip (Figure 4B; Figure S24). A current of 0.35 A was applied, reducing the probe body temperature by 20.0°C. Based on prior gel‐based calibration and direct measurements, the temperature drop at the tip was estimated at approximately 6.0°C (Figure 4D, top panel). This controlled cooling enabled reliable suppression of neural activity without thermal damage to the surrounding tissue, and no stimulus artifacts were detected in the raw traces.

Real‐time monitoring revealed a marked suppression in LC neuronal firing during the cooling period. Multi‐unit firing rates began to decline ∼1 s after activation of thermoelectric cooler, reaching maximal inhibition (∼80.0% reduction) within ∼6 s, and gradually returning to baseline during the rest phase (Figure 4D, middle panel). Importantly, this neural inhibition coincided with a reduction in pupil diameter, a physiological response linked to LC activity. Pupil constriction was observed during each cooling cycle and reversed during the off periods (Figure 4D, bottom panel and Figure S25). Previous studies have demonstrated a causal link between LC activation and pupil dilation, highlighting the LC's role in autonomic regulation [35, 36, 37, 38]. To explore this further, we simultaneously recorded spiking activity and pupil size in head‐fixed mice. While the overall trend showed cooling‐induced neuronal inhibition and concurrent pupil constriction, we also noted trial‐to‐trial variability in pupil responses. This variation may result from contributions of other neuromodulatory systems, such as cholinergic and serotonergic pathways, or from hormonal and systemic factors known to influence pupil dynamics [39] (Movie S3).

We quantitatively compared neuronal firing and pupil diameter before, during, and after cooling phases, summarized in histogram plots (Figure 4C,E). These histograms confirm the temporal correlation between LC inhibition and reduced pupil size, underscoring the functional efficacy of our cooling‐based modulation system. Additionally, action potentials from individual neurons located at various distances from the probe tip showed different amplitude profiles, further demonstrating the probe's sensitivity and spatial influence (Figure 4F–I).

**FIGURE 5:**
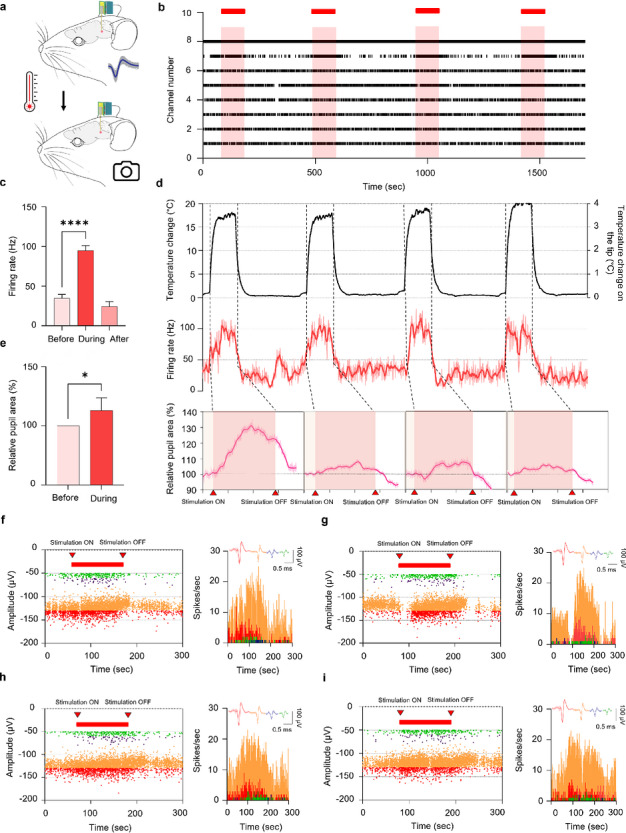
Heating modulation capability of the miniaturized bidirectional thermal stimulation system. (A) Schematic illustration of the experimental setup for applying localized heating and simultaneously recording neuronal activity and pupil dynamics *(n* = 1, where n is the number of animals). (B) Raster plot showing multi‐channel LC spike activity during repeated heating stimulation cycles (red bars indicate stimulation periods). (C) Quantification of LC neuron firing rates, highlighting enhanced excitatory responses before, during, and after heating stimulation. (*p* = 0.0001; *n* = 4, where *n* is the number of stimulations, *t* = 18.89). (D) Simultaneous recordings of temperature change (top), LC firing rate (middle), and normalized pupil area (bottom), revealing synchronized increases in neural firing and pupil dilation in response to heating. (E) Quantified pupil area before and during heating stimulation. (*p* = 0.0488; *n* = 4, where n is the number of stimulations, *t* = 2.465). (F–I) Firing rates of representative LC neurons during heating stimulation cycles. Insets show corresponding spike waveforms. The red line above the raster plots indicates the stimulation duration (3 cycles; 2 min ON and 4 min OFF per cycle). All statistical analyses were performed by the two‐tailed unpaired *t*‐test, and *p* < 0.05 was considered significant. ^*^ *p* < 0.05, ^**^ *p* < 0.01, ^***^ *p* < 0.001. ns: no statistical significance.

To demonstrate the bidirectional modulation capability of our cryoprobe system, we conducted heating experiments using the same ON/OFF protocol as cooling (Figure 5A). Current was carefully regulated to elevate temperature by ∼4°C–5°C − a safe range for neural tissue [40, 41]. During heating, LC neurons displayed a robust excitatory response, with multi‐unit firing rates increasing in ∼303.0% of recorded units (Figure 5D, middle panel and Figure S26). This excitatory modulation was further reflected in pupil dilation, which peaked in synchrony with the maximum temperature (Figure 5D, bottom panel and Figure S27). On average, pupil diameter increased by ∼1.3‐fold during heating and returned to baseline during rest phases (Movie S4). In parallel, the spike raster and single‐unit firing‐rate plots (Figure 5F–I) demonstrate consistent and reproducible excitatory responses in both proximal and distal neurons relative to the neural probe, with mean firing rates increasing approximately 2.5‐fold in proximal neurons and 1.5‐fold in distal neurons during stimulation compared to baseline.

Interestingly, neuronal firing rates remained elevated for ∼20.0 s after heating ceased, suggesting a prolonged excitatory effect on LC circuitry. This prolonged may reflect the activation of LC‐mediated sympathetic pathways and delayed recovery of neuronal excitability following thermal stimulation.

To quantify the temporal dynamics of thermal modulation, we analyzed the response latency during both cooling and heating stimulation. Response latency was defined by comparing the firing rate (FR) during stimulation with the baseline FR calculated over the 30 s period preceding stimulation onset. For cooling stimulation, the response latency was defined as the time point at which the firing rate of the recorded spike units decreased to 10.0% of the baseline level, while for heating stimulation, the latency corresponded to the time point at which the firing rate increased to 130.0% of the baseline level. Based on this criterion, the average response latency was approximately 3.11 s for heating stimulation and 5.7 s for cooling stimulation across stimulation trials (Figure S28).

Neuronal responses to thermal stimuli are known to vary substantially across brain regions and neuronal populations [42]. These effects are generally attributed to temperature‐dependent changes in membrane dynamics and ion channel kinetics, as well as the distribution of thermosensitive receptors. In the present study, mild heating of the LC region predominantly induced excitatory responses, which was further supported by population‐level analysis showing that all recorded neurons exhibited increased firing rates during stimulation, with an average ∼2–3‐fold elevation relative to baseline (Figure S29). In contrast, cooling consistently suppressed neuronal activity. Such bidirectional effects may arise from temperature‐dependent modulation of ion channel activity, intrinsic cellular properties, or circuit‐level interactions within the LC network.

Notably, waveform characteristics remained unchanged even after prolonged stimulation, indicating minimal thermal damage to neurons.

To provide direct experimental evidence, we performed histological analysis of brain tissue following thermal stimulation. Importantly, 4′,6‐diamidino‐2‐phenylindole (DAPI) and neuronal nuclei (NeuN) staining did not reveal clear evidence of neuronal loss or structural damage around the implantation site, even after repeated heating‐cooling cycles (Figure S30).

Qualitative evaluation revealed consistent distributions of DAPI‐positive nuclei and NeuN‐positive neurons across control, single‐cycle, and repeated‐cycle conditions. A slightly increased density of DAPI signal was observed near the probe track in all groups, indicating localized cellular accumulation associated with probe insertion. Correspondingly, NeuN‐positive neurons exhibited a spatial redistribution away from the implantation site rather than a reduction in signal intensity. This pattern was consistent across all conditions, suggesting that it arises primarily from mechanical insertion rather than thermal stimulation.

Overall, we did not observe additional neuronal loss or progressive tissue damage following repeated stimulation cycles, indicating that the applied thermal modulation does not induce significant cumulative tissue damage.

In summary, our miniaturized bidirectional thermal stimulation system enables precise, reversible modulation of LC activity in both inhibitory and excitatory directions. The observed pupil constriction during cooling and dilation during heating confirm a direct link between thermal LC modulation and autonomic output (Figures S31–S33). This bidirectional capability offers a powerful framework for probing the neural mechanisms underlying physiological regulation and behavior within a single experimental context.

## 3 Discussion

Here, we present a miniaturized bidirectional thermal stimulation system integrated with a neural recording electrode array, capable of precisely modulating brain activity through localized temperature control. This system represents a significant advancement in neuromodulation technology by incorporating a compact thermoelectric module based on the Peltier effect, enabling both excitatory and inhibitory stimulation through current reversal. Unlike conventional methods, our platform allows for dual‐mode functionality with high spatial and temporal precision, achieved through continuous temperature monitoring and a thin, thermally conductive interface.

A key advantage of this system lies in its ability to simultaneously manipulate and record neural activity while monitoring behavioral outputs in vivo. This dual capability allowed us to perform within‐subject comparisons of opposing neuromodulatory effects in the same brain region under tightly controlled conditions. We demonstrated that the system reliably modulated neural activity in the locus coeruleus (LC), a key noradrenergic nucleus involved in arousal and autonomic control. Bidirectional thermal stimulation induced clear and reversible changes in both spiking activity and pupil diameter – pupil constriction during cooling and dilation during heating – highlighting a direct causal relationship between LC activity and physiological responses.

This study addresses several longstanding challenges in neuromodulation. The system's miniaturization, coupled with a reduced insulation thickness, minimizes implant invasiveness while preserving efficient thermal confinement. Thermochromic visualization in brain‐mimicking gels confirmed the spatially restricted heat propagation around the probe tip, demonstrating precise control over the affected tissue volume and mitigating the risk of off‐target effects or thermal damage. Moreover, waveform stability and the absence of neuronal loss in immunohistological analysis observed over extended stimulation trials indicate that thermal delivery was safe and did not compromise neuronal integrity.

Compared to previous thermal stimulation devices, which have often been limited by bulkiness, poor thermal localization, or lack of recording integration, our system combines high‐resolution stimulation with high‐fidelity, real‐time neural and behavioral readouts. This versatility opens the door to deeper insights into the function of neuromodulatory circuits and how bidirectional control can shape brain state and behavior.

Importantly, this technology also holds strong translational potential. The system's precise and reversible modulation capabilities, along with its minimally invasive design, make it a promising candidate for therapeutic neuromodulation. Future work will extend the application of this system to animal models of neurological disorders, such as Parkinson's disease. By targeting motor‐related brain regions, we aim to explore the use of cryomodulation to alleviate motor symptoms and alter disease progression through circuit‐level interventions.

To further enhance long‐term performance, we plan to integrate a low‐impedance surface coating to improve chronic recording quality and signal stability during repeated thermal cycles. We note that the effective thermal modulation is influenced by implantation depth, as increases probe length within tissue enhances heat dissipation and reduces the achievable temperature gradient at the probe tip. Although our current design supports implantation up to ∼3.5 mm, the observed depth‐dependent attenuation suggests that extending this approach to centimeter‐scale depths, such as those required in larger animal models, would require further optimization. In particular, future structural refinements – such as replacing the current tubing with aerogel‐based thermal insulation and integration of a multistep Peltier module – will improve thermal confinement and enable further miniaturization of the device while preserving the thermal gradient. These modifications are expected to reduce tissue displacement and enhance the long‐term stability and viability of chronic implants.

In summary, our bidirectional thermal neural interface offers a highly precise, reversible, and safe approach for neuromodulation with integrated readout capabilities. By enabling localized control of both excitatory and inhibitory states within a single platform, this system provides a novel framework for dissecting the role of specific brain regions in behavior, with strong potential for both basic neuroscience and future clinical therapies.

## 4 Experimental Section

### 4.1 Fabrication of the Neural Probe Integrated With the Thermal Modulation System

The neural probe was fabricated through the previously developed processes. Briefly, we started with a 400‐nm‐thick silicon dioxide (SiO_2_) layer deposition on a 4‐inch silicon‐on‐insulator (SOI) wafer, followed by the deposition of a 20 nm titanium (Ti) adhesion layer and a 300 nm gold (Au) layer. Then the signal lines and electrode sites were formed using inductively coupled plasma (ICP) etching and the second 400‐nm‐thick insulation layer (SiO_2_) was deposited. The electrode site was patterned through the reactive ion etching (RIE) process, and Ti/Pt layers (20/150 nm) were deposited to form the platinum (Pt) microelectrode. Finally, the top and bottom sides of the wafer were defined and etched using the deep reative ion etching (DRIE) process to shape and release the shank. The fabricated neural probe was secured to a custom‐printed circuit board (PCB) with the metal matrix that manages the heat conduction to the implant using a thermal conductive grease (S6, IN CLOON YP‐S6, Korea) and a drop of quick‐setting adhesive (Loctite 401, LOCTITE, Germany). The contact pads of the neural probe were wire‐bonded to the exposed gold pads on the neural probe body of the PCB with a wire‐bonder (Kulicke and Soffa model 4526, Kulicke and Soffa, Republic of Singapore), and a flexible printed circuit (FPC) connector (503480‐1000, Molex, USA) was then soldered to the PCB.

To enhance the quality of neural signal recording, Pt black was electroplated onto the Pt electrodes of the neural probe. The plating solution was prepared by dissolving hexachloroplatinic acid hydrate (HCPA) in 0.025 N hydrochloric acid (HCl) with 0.025% lead acetate in deionized water. The neural probe was then submerged in this electroplating solution in a three‐electrode configuration, with a Pt wire—reference electrode and a silver/silver‐chloride (Ag/AgCl) wire (serving as the counter electrode). Pt black was deposited onto the recording Pt electrodes by applying an electrical potential of 0.25 V for 30 s using a potentiostat (PalmSens3, PalmSens, Netherlands).

### 4.2 Packaging of the Miniaturized Bidirectional Thermal Stimulation System

The fabricated probe was encapsulated with polyimide tubing (0.3 mm in inside diameter (+0.025 mm wall thickness), Goodfellow IM30‐TB‐000140‐02) such that around 0.7 mm of the probe shank was exposed. To ensure effective air insulation around the neural probe, the polyimide tube was sealed at both ends using a mix of polystyrene microparticles (74491‐5ML‐F, Sigma–Aldrich) and thermal epoxy (EPO‐TEK 320, Epoxy Technology, Inc., USA), creating a contained environment. First, a piece of the tube was carefully picked up using tape to facilitate handling. The shank of the probe was then gently inserted into the tube, ensuring that it was fully and securely positioned inside. Using optical fibers, a small cluster of dry beads, each approximately 20 µm in diameter, was transferred into the tube. It was important to ensure that a sufficient quantity of beads was positioned between each side of the probe and the inner wall of the tube, creating an even distribution.

Following this, the beads surrounding the shank were thoroughly wetted with an epoxy solution. This step required precise control of both the tube's position and the amount of epoxy applied, to maintain the integrity of the assembly and avoid excess adhesive. After allowing the epoxy to dry completely, the same procedure was repeated near the base of the shank, ensuring stability and proper adhesion throughout the entire length of the probe within the tube.

After a thorough inspection, an even layer of YP‐6 thermal compound was applied to the backside of the metal mesh PCB. The thermoelectric cooler (TEC) (CP1062‐268 Thermoelectric Peltier Module, 6.2 × 6.2 × 2.68 mm) was then carefully positioned and securely fixed on top of the thermal compound to ensure excellent thermal contact. Next, a lightweight heatsink (EPXNV9TM, 6.8 × 6 × 12 mm) was placed and precisely aligned on the system to enhance heat dissipation. To address high‐temperature operating conditions, a miniature fan (259‐2011‐ND, 17 mm x 17 mm) was also proposed to be incorporated.

### 4.3 Electrochemical Impedance

Electrochemical impedance spectroscopy (EIS) was performed to evaluate the electrochemical characteristics of the Pt black electrodes using a potentiostat (PGSTAT101, Metrohm AG, Herisau, Switzerland). Measurements were performed in a three‐electrode configuration, with the probe electrode as the working electrode, a platinum wire as the counter electrode, and an Ag/AgCl electrode as the reference. All measurements were conducted in phosphate‐buffered saline (PBS), with impedance recorded over a frequency range of 1 Hz–100 kHz.

### 4.4 Preparation of the Phantom for Thermochromic Test

To mimic the physical characteristics of the mouse brain, a 0.6% agarose gel was prepared. Agarose powder (A9539, Sigma–Aldrich, Germany) and deionized (DI) water were mixed in a beaker until the solution became fully transparent. After that, a thermochromic pigment in slurry form (Chameleon T series, Insilico Co., Ltd., Korea) was added, and the mixture was stirred for 1 min. The solution was then poured into a cube mold. It was left to cool down at room temperature to allow for gel formation. The gel was freshly made and immediately used in each experiment.

### 4.5 Thermochromic Performance

Each thermochromic hydrogel was engineered to respond to specific temperature thresholds. The hydrogel with a peak color change at 18°C was sensitive to cooling, changing color when the local temperature dropped to or below this point. Conversely, the hydrogel designed for 25°C responded to heating, transitioning in color when the temperature rose to or above the threshold. This configuration enabled clear visualization of both cooling and heating effects induced by the probe. As temperature varied, the hydrogel exhibited a gradual color transition, delineating the spatial extent of thermal stimulation.

To enable quantitative assessment, a calibration setup was established (Figures S9 and S10). Fresh hydrogel cubes were prepared, and a Peltier element was placed on the top surface, while a temperature sensor monitored the temperature along the sidewall. A MATLAB script was used to create a lookup table mapping color changes to corresponding temperature values recorded by the sensor. This calibration was performed for both heating and cooling cycles, generating a temperature‐color response curve.

Using this calibration, thermal effects near the neural probe were analyzed. To estimate the maximum temperature near the probe tip, temperature measurements under specific current conditions were used. The color change of pixels near the shank was compared to the calibration data to confirm the expected temperature variation. Based on the known pixel indices and their corresponding temperature values from the calibration setup, the approximate temperature distribution around the probe was determined. For example, a pixel index corresponding to a 4°C change in the calibration gel was assumed to reflect a similar temperature change near the probe.

### 4.6 Temperature Distribution Finite‐Element Analysis (FEA)

Finite‐element analysis was performed using COMSOL Multiphysics (COMSOL Inc., USA) to investigate the temperature distribution generated by the thermal neural probe in brain tissue. A 3D model was constructed to represent the probe and the surrounding tissue domain.

The simulations were carried out using the heat transfer in solids module. Brain tissue was modeled as a homogeneous medium with thermal properties based on literature values, including thermal conductivity = 0.5 W/m·K, density = 1040 kg/m^3^, and specific heat capacity = 3600 J/kg·K. For the polyimide tubing, the thermal conductivity was 0.12 W/m·K, and for the silicon (Si), it was 130 W/m·K. To approximate physiological conditions, the outer boundaries of the tissue domain were maintained at a constant temperature of 37.5°C. Heat transfer was assumed to occur primarily through conduction.

### 4.7 In Situ Temperature Measurements

An 80 µm‐diameter micro‐thermocouple (5SC‐TT‐K‐40‐72, Omega Engineering Inc., Norwalk) was attached to the packaged neural probe structure and fixed on the body and near the shank using quick‐setting adhesive (Loctite 401, LOCTITE, Germany). The thermocouple was aligned along the probe shank, with the sensor tip positioned near the exposed probe tip (∼20 µm separation). The assembled device was then implanted into the brain of an anesthetized mouse (AP = −1.8 mm; ML = 1.2 mm; DV = −3.2 mm).

### 4.8 In Vivo Electrophysiology

The Laboratory Animal Research Center, Korea University College of Medicine (KOREA‐2023‐0088) approved all animal procedures and experiments conducted in accordance with the ethical standards specified in the Animal Care and Use Guidelines. The miniaturized bidirectional thermal stimulation system was evaluated using adult male wild‐type mice (C57BL6; 9 weeks old). The mice were lightly anesthetized with isoflurane (approximately 1% during recording) before being positioned on a stereotaxic frame (David Kopf Instruments, USA). The procedure involved removing the skin and drilling the skull near the target area, guided by the Paxinos and Franklin atlas. The probe was then inserted into the drilled hole and carefully advanced to the locus coeruleus (LC) region (coordinates: bregma anteroposterior (AP) = −5.45 mm; mediolateral (ML) = 0.8 mm; dorsoventral (DV) = −3.5 mm).

Temperature stimulation was applied through repetitive ON/OFF cycles (2 V, 60/120/240 s), with current adjustments controlling the stimulation area. To ensure accurate temperature monitoring and thermal stability, a digital multimeter was used to continuously track the device's operational temperature during stimulation.

Pupil tracking was assessed by comparing the pupil size after LC stimulation to the resting state, with measurements recorded using a standard camera. Neural signals were captured, amplified, and digitized with the Intan RHD 2000 EVALUATION BOARD (Version 1.0, Intan Technologies). Electrical signals were processed through the device using a notch filter (60 Hz) and a bandpass filter (1–300 Hz), with data collected from 8 electrodes at a sampling rate of 20 kS/s. The raw data was analyzed using a custom MATLAB spike‐sorting algorithm, which semi‐automatically sorted neural signals and identified stimulation periods. The sorted signals were visualized in raster and bar plot to show firing rates over time. Statistical significance was performed using Student's *t*‐tests with GraphPad Prism (GraphPad Software Inc., USA).

### 4.9 Pupil Tracking

Pupil size was estimated using a MATLAB script (MathWorks, R2018a). First, each frame was converted to grayscale and smoothed using a Gaussian filter (with a sigma value of 2) to reduce noise. The pupil was then segmented from the background using adaptive thresholding, followed by inversion to highlight the pupil area against the darker background. Pupil area was estimated by counting the number of non‐black pixels within a manually selected region of interest (ROI). A rectangular ROI surrounding the pupil was defined in the first frame of each video. Each frame was then cropped to this ROI and converted to grayscale. Pixels with intensity values below a defined threshold (set to 50) were considered part of the pupil. The number of these dark pixels was computed for each frame and defined as the pupil area. Time stamps were recorded using the frame index and video frame rate. These values were exported to Excel for further analysis and plotted as pupil area over time. ROI boundaries were overlaid on each frame during analysis for visual confirmation of pupil tracking.

### 4.10 Immune Tissue Response

To evaluate tissue response following thermal stimulation, three conditions were examined: control (probe insertion only), a single stimulation cycle, and five repeated cycles. Each cycle consisted of four cooling (2 min ON, 4 min OFF) and four heating (2 min ON, 4 min OFF) phases. In repeated stimulation experiments, this sequence was applied five times with a 10 min interval between cycles.

Following stimulations, deeply anesthetized adult male wild‐type mice (C57BL6; 9 weeks old) were perfused with phosphate‐buffered saline (PBS), followed by 4% (w/v) paraformaldehyde (PFA). The brains were carefully extracted and soaked in 4% PFA at 4°C for 24 h. Subsequently, samples were cryoprotected in 20 and 30% sucrose solutions in PBS at 4°C until fully equilibrated.

The brains were sliced in the horizontal plane into 30 µm‐thick slices using a cryostat. Immediately after sectioning, the slices were directly mounted onto glass slides and allowed to adhere. The sections were rinsed in PBS and incubated in a blocking solution containing 0.1% (v/v) Triton X‐100 in PBS for 1.5 h at room temperature.

For cellular and neuronal labeling, the sections were incubated with primary antibodies against neuronal nuclei (NeuN, 1: 1,000, 266 004, Synaptic Systems) in PBS at 4°C overnight. Followed by PBS washing, each slice was incubated with appropriate fluorescent secondary antibody (donkey anti‐Guinea Pig conjugated Alexa Fluor 488, 1: 500, Abcam) for 1.5 h at room temperature. Nuclear staining was performed using 4′,6‐diamidino‐2‐phenylindole (DAPI 1:1,000, D1306, Invitrogen) in PBS for 5 min at room temperature.

After the final washing steps, the brain sections were gently rinsed using PBS and maintained on glass slides for imaging. Fluorescence images were acquired using a confocal microscope (STELLARIS, Leica Microsystems, Danaher Corporation, USA). For quantitative analysis, fluorescence images were processed using ImageJ.

### 4.11 Statistical Analysis

All statistical analyses were performed using GraphPad Prism (GraphPad Software, USA). Data were inspected prior to analysis, and no data points were excluded.

Individual data points are presented unless otherwise specified. The sample size (*n*) for each analysis is indicated in the corresponding figure captions. In this study, *n* represents either the number of independent stimulation events or the number of animals, depending on the experiment. For neural activity and pupil measurements, repeated stimulation cycles within the same animal were analyzed as independent trials due to temporal separation and stable baseline conditions between cycles.

Statistical comparisons between groups were performed using two‐tailed unpaired Student's *t*‐tests. A significance level of α = 0.05 was used for all analyses. Statistical significance was defined as *p* < 0.05. The following thresholds were used to denote significance levels in the figures: *p* < 0.05 (^*^), *p* < 0.01 (^**^), *p* < 0.001 (^***^), and *p* < 0.0001 (^****^), while “ns” indicates no statistical significance. Exact *p*‐values are reported where applicable.

No multiple comparison corrections were applied, as comparisons were conducted between predefined experimental conditions. Statistical details, including the number of samples and tests used, are provided in the corresponding figure legends.

## Author Contributions

Z.N. performed most of the experiments, analyzed the data, prepared the figures, and wrote the manuscript. K.K. conceived the project, designed the study, and prepared the initial scripts. Z.N. and K.K. jointly contributed to the realization of the idea, designed and fabricated the device, and performed preliminary in vivo experiments. W.C.S. participated in discussion on the in‐vivo experiments, provided suggestions for data visualization. K.K and S.L were involved in preparing the histology experiments. J.W. was involved in the in vivo experiments preparation. I.‐J.C. discussed the results, provided comments, and wrote the manuscript. All authors reviewed the manuscript.

## Conflicts of Interest

The authors declare no conflicts of interest.

## Supporting information




**Supporting File 1**: advs75671‐sup‐0001‐SuppMat.docx.

**Supporting File 2**: advs75671‐sup‐0002‐Movie S1.mp4.

**Supporting File 3**: advs75671‐sup‐0003‐Movie S2.mp4.

**Supporting File 4**: advs75671‐sup‐0004‐Movie S3.mp4.

**Supporting File 5**: advs75671‐sup‐0005‐Movie S4.mp4.

## Data Availability

The authors declare that all data supporting the findings of this study are provided within the main text and the supplementary information files.
