# Supplementary material for: Miniaturized Bidirectional Thermal Stimulation System Integrated With an Electrode Array for Recording Neural Activities
Source: Adv Sci (Weinh). 2026 May 20:e22077. Online ahead of print. doi: 10.1002/advs.202522077 (PMC13335964; doi:10.1002/advs.202522077)
Supplement: Supplementary file 1 — Supporting File 1: advs75671‐sup‐0001‐SuppMat.docx. [file ADVS-9999-e22077-s004.docx]

Supporting Information

**Miniaturized Bidirectional Thermal Stimulation System Integrated with An Electrode Array for Recording Neural Activities**

*Zoia Naumkina^1†^, Kanghwan Kim^2†^, Wesley Charles Smith^2^, Keuntae Kim^3^, Shinwoo Lee^4^, Jiwan Woo^5^, Il-Joo Cho^1*^*

^†^These authors contributed equally to this work

*Corresponding author

70 μm

14 μm

700 μm

30 μm

220 μm

325 μm

100 μm

**Figure S1.** **Detailed dimensions of the fabricated neural probe.**

**Figure S2. Fabrication process flow of the thermal neural probe.** A cross-sectional view of A-A’ **A** to **J** used to present the detailed fabrication steps at the point of view for electrode location. **A,** Deposition of the first SiO₂ insulation layer on a silicon substrate. **B,** Deposition and patterning of Ti/Au signal lines. **C,** Deposition of the second SiO₂ layer for insulation of signal lines. **D,** Etching of the SiO₂ layer to expose electrode sites and wire bonding pads using reactive ion etching (RIE). **E-F,** Patterning and deposition of Ti/Pt on the electrode sites for neural recording. **G,** Reactive ion etching (RIE) of SiO₂ insulation layers. **H,** Deep reactive ion etching (DRIE) of the top silicon layer to define the probe structure. **I** Backside etching of the silicon substrate to release the probe shank. **J** Released neural probe.

Silicon

SiO_2_

Au

SiO_2_

Pt

A

A’

**A**

**B**

**C**

**D**

**E**

**F**

**G**

**H**

**I**

**J**

A

A’

A

A’

Signal lines

1^st^ insulation layer

2^nd^ insulation layer

Pt electrode

A

A’

A

A’

300 μm

220 μm

25 μm

Si

4000 Å (SiO_2_)

200 Å/3000 Å (Ti/Au)

200 Å/1500 Å (Ti/Pt)

3 μm (Black Pt)

**Figure S3. Cross-sectional schematic showing thickness and material composition of the probe shank with electrodes.**

**Figure S4. Electrical characterization of the neural probe electrodes. A,** Photographs showing the neural probe shank and Pt black electrode after electroplating. **B,** The electrochemical impedance spectroscopy (EIS) in a range from 0 to 100 kHz at 32 electrodes after Pt black electroplating.

Neural probe shank

Temperature sensor

**A**

**B**

**C**

**D**

Metal-layer integrated PCB

Peltier element

Heat sink

Thin layer of thermal paste

5 mm

5 mm

5 mm

5 mm

**Figure S5. Representative photos of the packaged system**. **A,** Packaged neural probe with a fixed temperature sensor and polyimide tube, attached to the PCB. **B,** Backside view of the PCB, featuring a metal layer for improved thermal conductivity and structural support. **C,** Placement of the Peltier element on the PCB with a thin layer of thermal paste for efficient heat transfer. **D,** Integration of a heat sink on the Peltier element using glue drops over the edges of the Peltier element, with an additional layer of thermal paste.

**Figure S6. Temperature measurement at the neural probe body and tip.** A temperature sensor was placed on the probe tip to monitor the local heating/cooling at the shank end, while another sensor was positioned on the probe body to track the overall device temperature during each thermal stimulation cycles.

Temperature sensor

Neural probe tip

Temperature sensor on the body

5 mm

**Figure S7. Cooling cycle estimation on thermochromic hydrogel.** Photograph of the neural probe places on a cold-sensitive thermochromic hydrogel with body-fixed temperature sensor. As the driving current increased from 0.1 to 0.35 A, the hydrogel exhibited a progressive color change, indicating localized cooling at the probe tip.

0.3 A

0.15 A

0.2 A

0.25 A

0.35 A

0.1 A

Temperature sensor

Hydrogel

**Figure S8. Heating cycle estimation on thermochromic hydrogel.** Sequential photographs showing the neural probe in contact with a heat-sensitive thermochromic hydrogel. As the input current increased from 0.1 A to 0.3 A, the hydrogel progressively shifted in color around the probe tip, reflecting localized heating induced by the Peltier element.

0.25 A

0.1 A

0.15 A

0.2 A

0.3 A

Hydrogel

Temperature sensor

**Figure S9. Heating performance of the neural probe in thermochromic gel under stimulation (0.35 A). A,** Photograph and corresponding false-color thermal map demonstrate localized heating around the probe tip during current-driven stimulation. **B,** Spatial temperature distribution extracted along the probe axis reveals both the peak temperature elevation and lateral spread.

**-800**

**2**

**0**

**Temperature change (°C)**

**-600**

**-200**

**0**

**200**

**400**

**Spatial distribution of temperature (μm)**

**-400**

**600**

**800**

**4**

**6**

**8**

**A**

**B**

200 μm

200 μm

**Figure S10. Cooling cycle on thermochromic hydrogel using a direct Peltier element. A,** Sequential image showing the temperature-dependent color change of the thermochromic hydrogel during the cooling cycle. The Peltier element with a heat sink is placed in contact with the hydrogel, and a temperature sensor monitors the thermal response. The numbered steps correspond to different time points in the cooling process. **B,** Temperature change over time, demonstrating the thermal response of the hydrogel. The plot tracks the cooling and recovery cycle, with numbered points matching the images in **A**.

Temperature sensor

**- 10**

**- 15**

**- 5**

**0**

**Time (sec)**

**0**

**50**

**100**

**150**

**Temperature change (°C)**

Temperature sensor on the body

**A**

**1**

**2**

**3**

**4**

**7**

**6**

**5**

**8**

**1**

**2**

**3**

**4**

**5**

**6**

**7**

**8**

Temperature sensor

Peltier element

Heat sink

**B**

**Figure S11. Estimation of the heating cycle on the thermochromic hydrogel using direct Peltier element. A,** Sequential image showing the temperature-dependent color change of the thermochromic hydrogel during the heating cycle. The Peltier element with a heat sink is placed in contact with the hydrogel, and a temperature sensor monitors the thermal response. **B,** Temperature change over time, demonstrating the thermal response of the hydrogel. The plot tracks the heating and recovery cycle, with numbered points matching the images in **A**.

**10**

**Temperature change (°C)**

**- 5**

**0**

**Time (sec)**

**5**

**0**

**50**

**100**

**150**

**A**

**1**

**2**

**3**

**4**

**7**

**6**

**5**

**8**

Temperature sensor

Peltier element

Heat sink

**1**

**2**

**3**

**4**

**5**

**6**

**7**

**8**

**B**

**Figure S12. Quantification of localized temperature change within 200 μm of the probe tip under varying stimulation currents. A,** Cooling cycles**. B,** Heating cycles.

**0.1**

**0.15**

**0.2**

**0.25**

**0.3**

**Current (A)**

**0.35**

**4**

**0**

**8**

**2**

**6**

**Temperature change near the tip (°C)**

**A**

**B**

**Cooling**

**Current (A)**

**4**

**0**

**8**

**2**

**6**

**Temperature change near the tip (°C)**

**Heating**

**0.1**

**0.15**

**0.2**

**0.25**

**0.3**

**0.35**

**Figure S13. COMSOL finite-element analysis of the temperature distribution inside the brain tissue.** **A**, 3D revolved geometry view of the neural probe inserted into brain tissue, showing the spatial temperature distribution during cooling stimulation. **B**, 3D simulation of spatial temperature distribution during heating stimulation. Each inset illustrates the front position of the cryoprobe.

**Figure S14. COMSOL analysis of temperature distribution at varying probe insertion depths. A,** Cooling profiles at 0.7 mm, 1 mm, and 3.5 mm. **B,** Heating-induced temperature profiles at corresponding insertion depths.

**Figure S15. COMSOL-derived temperature profiles at varying probe insertion depths. A,** Cooling temperature profiles extracted at defined distances from the probe tip for insertion depths of 0.7 mm, 1 mm, and 3.5 mm. **B,** Heating-induced temperature profiles at corresponding insertion depths.

**Figure S16. Temperature monitoring setup for neural probe experiments. A,** Schematic illustration of sensor placement on the probe body and at the probe tip within the brain. **B,** Optical images showing probe geometry and sensor positioning near the tip.

**Figure S17. Temperature profiles recorded using an external thermocouple sensor inside living brain tissue. A,** Temperature changes during cooling stimulation**. B,** Heating-induced temperature changes.

Intan RHD2116

Recording IC

Intan RHD2000

Interface PCB

x32

To PC

Electrical activity

**+**

**-**

Multimeter

To PC

Temperature monitoring

Power supply

Operating current

0.1 … 0.35 A

Camera

To PC

Pupil monitoring

Recording electrodes

Peltier element

**Figure S18. Schematic illustration of the system operation.** A box with the red-dashed line indicates the Peltier element, which is used for bidirectional thermal stimulation of brain tissue. The Peltier element operates with an applied voltage (1.4 V) supplied by a power source, while a multimeter monitors the temperature changes through the integrated temperature sensor. A box with the green-dashed line shows the recording electrodes integrated on the probe shank. The probe includes 32 recording sites for electrical activity measurements. The signals from the electrodes are transmitted to the Intan RHD2116 recording IC and then transferred via the RHD2000 interface PCB to the PC for data acquisition. For behavioral monitoring, a camera is positioned to record pupil dynamics, and the video stream is also transferred to the PC.

**Figure S19. Collation of the firing rate response during the cooling stimulation of the cerebellum area. A,** Neural activity recorded from the cerebellum region showing transient suppression of firing rate (blue trace) during repeated cooling cycles. **B,** Temperature profile measured at the probe body (black trace), indicating periodic decreases of up to ~17 °C that correspond to the reductions in firing rate. The alignment of both traces demonstrates a reversible modulation of neural activity tightly coupled to cooling stimulation.

**A**

**B**

**0**

**-15**

**- 10**

**Firing rate (Hz)**

**Temperature change (°C)**

**- 5**

**Time (sec)**

**0**

**20**

**10**

**40**

**30**

**50**

**0**

**1000**

**1500**

**500**

**0**

**2000**

**-20**

**#1**

**#2**

**#3**

**#4**

**1000**

**1500**

**500**

**2000**

**Figure S20. Comparison of firing rate modulation during cooling and heating stimulation. A,** Firing rate reduction during cooling stimulation. Bar plot showing a temperature-dependent decrease in neuronal firing rate as the temperature drop increases. **B,** Firing rate enhancement during heating stimulation. Bar plot illustrating a clear increase in neuronal firing rate with greater temperature elevation.

**A**

**Firing rate (Hz)**

**Temperature change (Δ°C)**

**Firing rate (Hz)**

**Temperature change (Δ°C)**

**B**

**Cooling**

**Heating**

**0**

**10**

**5**

**15**

**-3.8**

**-5.2**

**-5.9**

**-6.8**

**3.2**

**4.3**

**6.7**

**7.6**

**0**

**100**

**50**

**150**

**200**

**Figure S21. Representative plot of the recorded neural signal amplitude under different powers of cooling stimulation. A-D,** Scatter plots displaying the neural signal amplitude (µV) over time across different cooling power levels. Each point represents a detected spike, with amplitude changes illustrating neural activity variations.

**A**

**B**

**C**

**D**

**Time (sec)**

**Amplitude (μV)**

**-150**

**-50**

**-100**

**0**

**50**

**100**

**0**

**#1**

**Time (sec)**

**Amplitude (μV)**

**-150**

**-50**

**-100**

**0**

**50**

**100**

**0**

**#2**

**Time (sec)**

**Amplitude (μV)**

**-150**

**-50**

**-100**

**0**

**50**

**100**

**0**

**#3**

**Time (sec)**

**Amplitude (μV)**

**-150**

**-50**

**-100**

**0**

**50**

**100**

**0**

**#4**

**Figure S22. Correlation between neural firing rate and local temperature elevation during heating stimulation.** The upper red trace (**A**) shows the firing rate (Hz) of neural activity recorded in the cerebellum, while the lower black trace (**B**) depicts the corresponding temperature increase (°C) at the probe tip. Periodic heating cycles induced reproducible elevations in firing rate that closely followed the temperature dynamics.

**A**

**B**

**Firing rate (Hz)**

**0**

**20**

**10**

**25**

**30**

**Temperature change (°C)**

**100**

**150**

**50**

**#1**

**#2**

**#4**

**#5**

**Time (sec)**

**1000**

**1500**

**500**

**0**

**2000**

**1000**

**1500**

**500**

**0**

**2000**

**Figure S23. Representative plot of the recorded neural signal amplitude during different power of heating stimulation. A-D,** Scatter plots showing the neural signal amplitude (µV) over time across varying heating power levels. Each point represents a detected spike, with amplitude changes reflecting neural activity fluctuations.

**A**

**B**

**C**

**D**

**Time (sec)**

**Amplitude (μV)**

**-150**

**-50**

**-100**

**0**

**50**

**100**

**0**

**-200**

**#1**

**Time (sec)**

**Amplitude (μV)**

**-150**

**-50**

**-100**

**0**

**50**

**100**

**0**

**-200**

**#2**

**Time (sec)**

**Amplitude (μV)**

**-150**

**-50**

**-100**

**0**

**50**

**100**

**0**

**-200**

**#3**

**Time (sec)**

**Amplitude (μV)**

**-150**

**-50**

**-100**

**0**

**50**

**100**

**0**

**-200**

**#4**

**Figure S24. Raster plots of neural activity recorded across multiple channels during four distinct cooling stimulation cycles. A-D,** Raster plots showing the spike activity across different recording channels over time. Each row represents a channel, and each black tick marks an individual spike event. The blue bars indicate the duration of four separate cooling stimulation cycles (**A**: cycle #1, **B**: cycle #2, **C**: cycle #3, **D**: cycle #4). The neural activity patterns demonstrate the effect of cooling on neuronal firing across multiple trials.

**Time (sec)**

**100**

**150**

**50**

**200**

**Time (sec)**

**550**

**600**

**500**

**650**

**Time (sec)**

**1000**

**1050**

**950**

**1100**

**Time (sec)**

**1500**

**1550**

**1450**

**1600**

**Stimulation cycle #1**

**Stimulation cycle #2**

**Stimulation cycle #3**

**Stimulation cycle #4**

**A**

**B**

**C**

**D**

**6**

**4**

**2**

**0**

**8**

**10**

**0**

**Channel number**

**6**

**4**

**2**

**0**

**8**

**10**

**Channel number**

**6**

**4**

**2**

**0**

**8**

**10**

**Channel number**

**6**

**4**

**2**

**0**

**8**

**10**

**Channel number**

**Figure S25. Pupil dilation in response to cooling stimulation of LC. A-D,** Bar plots depicting mean pupil diameter (in pixels) measured before, during, and after cooling stimulation. Each subplot (**A**, **B**, **C**, **D**) corresponds to different experimental trials.

**Mean pupil diameter (pixel)**

**Before**

**During**

**After**

**Mean pupil diameter (pixel)**

**Before**

**During**

**After**

**Mean pupil diameter (pixel)**

**Before**

**During**

**After**

**Before**

**During**

**After**

**Mean pupil diameter (pixel)**

**A**

**B**

**C**

**D**

**100**

**150**

**50**

**0**

**100**

**150**

**50**

**0**

**100**

**150**

**50**

**0**

**100**

**150**

**50**

**0**

**Figure S26. Raster plots of neural activity recorded across multiple channels during four distinct heating stimulation cycles. A-D,** Raster plots illustrating spike activity across different recording channels over time. Each row represents a channel, with black ticks marking individual spike events. The red bars indicate the duration of four separate heating stimulation cycles (**A**: cycle #1, **B**: cycle #2, **C**: cycle #3, **D**: cycle #4). The neural activity patterns reveal the effect of heating on neuronal firing across multiple trials.

**Time (sec)**

**100**

**150**

**50**

**Time (sec)**

**450**

**550**

**600**

**500**

**Time (sec)**

**1000**

**1100**

**950**

**Time (sec)**

**1400**

**1500**

**1550**

**1450**

**Stimulation cycle #1**

**Stimulation cycle #2**

**Stimulation cycle #3**

**Stimulation cycle #4**

**D**

**6**

**4**

**2**

**0**

**8**

**10**

**Channel number**

**A**

**6**

**4**

**2**

**0**

**8**

**10**

**Channel number**

**C**

**6**

**4**

**2**

**0**

**8**

**10**

**Channel number**

**B**

**6**

**4**

**2**

**0**

**8**

**10**

**Channel number**

**200**

**650**

**0**

**1050**

**Figure S27. Pupil constriction in response to heating stimulation of LC. A-D,** Bar plots showing mean pupil diameter (in pixels) measured before, during, and after heating stimulation. Each subplot (**A**, **B**, **C**, **D**) represents different experimental trials.

**Before**

**During**

**After**

**Before**

**During**

**After**

**Before**

**During**

**After**

**Before**

**During**

**After**

**Mean pupil diameter (pixel)**

**Mean pupil diameter (pixel)**

**Mean pupil diameter (pixel)**

**Mean pupil diameter (pixel)**

**A**

**B**

**C**

**D**

**0**

**100**

**50**

**150**

**0**

**100**

**50**

**150**

**0**

**100**

**50**

**150**

**0**

**100**

**50**

**150**

**Figure S28. Comparison of the representative neuron response latency during each stimulation event. A,** Response latency profile during cooling stimulation. **B,** Response latency profile during heating stimulation.

**Figure S29.Comparison of changes in single-unit firing rates across stimulation cycles during heating. A-D,** Firing rate profiles for the 1^st^-4^th^ stimulation cycles before (baseline) and during stimulation**.**

**Single stimulation**

**5 stimulation cycles**

**Merged**

**DAPI**

**NeuN**

**Control**

**A**

**0**

**100**

**200**

**300**

**0**

**50**

**100**

**Fluorescent intensity (a.u.)**

**Distance (μm)**

**0**

**100**

**200**

**300**

**0**

**50**

**100**

**Distance (μm)**

**0**

**100**

**200**

**300**

**0**

**50**

**Distance (μm)**

**100**

**Green: NeuN**

**Blue: DAPI**

**Green: NeuN**

**Blue: DAPI**

**Green: NeuN**

**Blue: DAPI**

**B**

**Figure S30. Immune response around the thermal neural probe following stimulation.**
**A,** Representative confocal fluorescence images of horizontal brain slices (30 μm thickness) showing neuronal nuclei (NeuN, green) and cell nuclei (DAPI, blue) under three conditions: control, single stimulation, and five stimulation cycles. The dashed circle indicates the probe implantation site (scale bar – 100 µm). **B,** Corresponding line intensity profiles extracted along the red dashed line in (**A**), showing fluorescence intensity of NeuN (green) and DAPI (blue) as a function of distance from the probe center.

**Figure S31. Representative pupil images showing dilation during cooling stimulation.** Pupil images captured before (0 sec), during (30-120 sec), and after (150 sec) cooling stimulation. The pupil boundary is highlighted (dashed circle) to illustrate dynamic changes in pupil size. A clear dilation is observed during the cooling period, followed by recovery to baseline after stimulation (*n = 1, where n is a number of animals per stimulation condition*).

**Figure S32. Pupil images showing constriction during heating stimulation.** Representative pupil images recorded before (0 sec), during (30–120 sec), and after (150 sec) heating stimulation. The pupil outline (dashed circle) highlights progressive constriction in response to heating, followed by a return toward baseline size once the stimulation ceased. (*n = 1, where n is a number of animals per stimulation condition*).

**Figure S33. Additional examples of pupil responses during cooling and heating stimulation. A,** Cooling-induced pupil responses at 0.35 A stimulation current. **B,** Heating-induced pupil responses at 0.2 A current. Images are shown before (0 s), during (30–120 s), and after (150 s) stimulation. Each colored dashed box represents an individual animal, and dashed circles indicate pupil boundaries (*n = 2, where n is a number of animals per stimulation condition*).
**Movie S1**. Visualization of localized cooling using thermochromic hydrogel.

**Movie S2.** Visualization of localized heating using thermochromic hydrogel.

**Movie S3.** Pupil dynamics of a mouse recorded during cooling stimulation using the bidirectional thermal modulation system.

**Movie S4.** Pupil dynamics of a mouse recorded during heating stimulation using the bidirectional thermal modulation system.
